# The Alternative RelB NF-kB Subunit Exerts a Critical Survival Function upon Metabolic Stress in Diffuse Large B-Cell Lymphoma-Derived Cells

**DOI:** 10.3390/biomedicines10020348

**Published:** 2022-02-01

**Authors:** Stéphanie Nuan-Aliman, Didier Bordereaux, Catherine Thieblemont, Véronique Baud

**Affiliations:** 1 NF-kappaB, Différenciation et Cancer, Université de Paris, 75006 Paris, France; stephanie.nuan@gmail.com (S.N.-A.); bordereauxdidier@gmail.com (D.B.); catherine.thieblemont@aphp.fr (C.T.); 2 Hémato-Oncologie, APHP Hôpital Saint-Louis, 75010 Paris, France

**Keywords:** NF-κB, RelB, lymphoma, DLBCL, metabolism, apoptosis

## Abstract

Diffuse large B-cell lymphoma (DLBCL) is the most common non-Hodgkin lymphoma in adults and reveals distinct genetic and metabolic signatures. NF-κB transcription factor family is involved in diverse biological processes enabling tumor development and resistance to anticancer-therapy through activation of its two main pathways, the canonical and the alternative NF-κB pathways, the main actor of the latter being the RelB NF-kB subunit. RelB DNA binding activity is frequently activated in DLBCL patients and cell lines. RelB activation defines a new DLBCL subgroup with dismal outcome upon immunochemotherapy, and RelB confers DLBCL cell resistance to DNA damage. However, whether RelB can impact on DLBCL cell metabolism and survival upon metabolic stress is unknown. Here, we reveal that RelB controls DLBCL oxidative energetic metabolism. Accordingly, RelB inhibition reduce DLBCL mitochondrial ATP production, and sensitizes DLBCL cells to apoptosis induced by Metformin and L-asparaginase (^®^Kidrolase), two FDA approved antimetabolic drugs targeting mitochondrial metabolism. RelB also confers DLBCL cell resistance to glutamine deprivation, an essential amino acid that feeds the TCA cycle. Taken together, our findings uncover a new role for RelB in the regulation of DLBCL cell metabolism and DLBCL cell survival upon metabolic stress.

## 1. Introduction

NF-κB is a family of transcription factors that are key players in cell survival, immune and inflammatory responses [1,2,3]. In mammals, it is composed of five structurally related members forming homo- or heterodimers: RelA (also known as p65), RelB, cRel (Rel), NF-κB1 (p50 and its precursor p105) and NF-κB2 (p52 and its precursor p100) [4]. Its activity is regulated through two main signaling cascades: the classical or canonical NF-κB pathway, in which mainly participate RelA and/or c-Rel containing complexes; and the alternative or non-canonical NF-κB pathway that leads to the activation of RelB containing dimers [5,6,7].

Diffuse large B-cell lymphoma (DLBCL) is the most common type of non-Hodgkin lymphoma (NHL) in adult [8]. The addition of the anti-CD20 monoclonal antibody Rituximab (R) to the standard of care CHOP regimen (cyclophosphamide, doxorubicin, vincristine, prednisone) was a major advance in the front-line treatment [9]. However, up to 40% of patients will ultimately relapse or progress and die [10,11]. DLBCL is a highly heterogeneous disease, and major efforts have been made to characterize such heterogeneity. First, gene expression profiling identified two prominent cell-of-origin (COO) DLBCL subtypes, the germinal center B-cell-like (GCB), and the activated B-cell-like (ABC), while 10–20% of cases remain unclassified [12]. ABC DLBCL are associated with worse outcome among patients treated with immunochemotherapy [13]. In another transcriptomic approach, a consensus cluster classification (CCC) identified three subgroups: the BCR/proliferation cluster (BCR-DLBCL) displaying up-regulation of genes encoding B-cell receptor (BCR) signaling components, the OxPhos cluster (OxPhos-DLBCL) significantly enriched in genes involved in mitochondrial oxidative phosphorylation (OxPhos), and the host response (HR) tumors characterized by a brisk host inflammatory infiltrate [14]. The so-called OxPhos-DLBCL subset was functionally characterized by a non-functional BCR signaling and an increased mitochondrial metabolism required for this DLBCL subset survival [15,16]. In recent years, the development of the next-generation sequencing (NGS) technologies revealed recurrent genetic alterations and allowed the subclassification of DLBCL into several genetic clusters [17,18]. Frequent mutations in regulators of the classical NF-κB pathway (MYD88, TNFAIP3, CD79A/B, CARD11) are recognized as a hallmark of ABC DLBCL patients [19,20,21,22,23], and inhibition of the classical NF-κB pathway induces cell death in ABC DLBCL cell lines [24]. Recently, our laboratory has uncovered that the alternative RelB NF-κB subunit is frequently activated in DLBCL patients and cell lines, independently of their ABC or GCB subtypes [25]. Most importantly, RelB activity predicted worse overall survival upon immunochemotherapy [25]. Further, we shed light on a new role for RelB in promoting DLBCL cell survival upon treatment with genotoxic agents [25]. Beside the significant advances in the understanding of RelB function in DLBCL, whether RelB activation might be connected to the OxPhos metabolic status of DLBCL cells is still unknown.

Cancer cells exhibit metabolic reprogramming in order to meet their high bioenergetics and biosynthetic demands [26]. Such deregulation of cellular energetics has now been recognized as a hallmark of cancer [27]. Metabolic adaptation associated with tumoral cells was first linked to an increase in aerobic glycolysis, a phenomenon described as the “Warburg effect” [28,29]. However, it is now recognized that deregulation of mitochondrial oxidative phosphorylation also plays a crucial role for tumor cell survival and proliferation [28,30]. A link between the classical RelA NF-κB subunit and the ability of cancer cells to reprogram their metabolism has been reported in recent years [31,32,33]. In contrast, to our knowledge the role of the alternative RelB NF-κB subunit in cancer cell metabolism has not yet been reported, and whether it may impact on DLBCL OxPhos status for survival remains an unanswered question.

In the study presented here, we reveal that the alternative RelB NF-κB subunit impacts on DLBCL cell energy homeostasis, enhancing OxPhos energy metabolism. We also find that RelB drives resistance of OxPhos-DLBCL cells to apoptosis induced by mitochondrial metabolic stress upon treatment with either Metformin or L-asparaginase (^®^Kidrolase), two approved anti-metabolic drugs, or glutamine deprivation. Moreover, we show that RelB activation in OxPhos-DLBCL cells is associated with increased expression of a subset of NF-κB target genes involved in oxidative/metabolic stress response, and survival upon antimetabolic treatment. Taken together, our findings uncover a new role for RelB in the regulation of DLBCL cell metabolism and DLBCL cell survival upon metabolic stress.

## 2. Materials and Methods

### 2.1. Antibodies and Reagents

The antibodies were purchased from Santa Cruz Biotechnology (RelB C-19), Merck (β-actin) and Cell Signaling Technology (Danvers, MA, USA) (cleaved caspase 3). The SYK inhibitor R406 was purchased from Santa Cruz Biotechnology (Santa Cruz, CA, USA). Metformin, 2-deoxy-D-glucose (2-DG) was purchased from Merck (Darmstadt, Germany), and L-asparaginase (^®^Kidrolase) was a kind gift from Isabelle Madelaine-Chambrin and Nathalie Jourdan (Service de Pharmacie Centrale Hôpital Saint-Louis).

### 2.2. Human DLBCL Cell Lines and Culture Conditions

MD901 and SUDHL-4 DLBCL cell lines were obtained from José Angel Martinez-Climent (Centro de Investigación Médica Aplicada, Pamplona, Spain). Cells were grown in RPMI-1640 Glutamax medium supplemented with 10% heat-inactivated fetal bovine serum (HyClone-Cytiva, Marlborough, MA, USA), 100 U/mL penicillin, and 100 mg/mL streptomycin (Life Technologies, Carlsbad, CA, USA).

For glutamine or glucose deprivation experiments, MD901 DLBCL cells were grown in RMPI 1640 without either glutamine or glucose or containing both glutamine and glucose and supplemented as above.

### 2.3. Lentiviral Production and Transduction

Production of infectious recombinant lentiviruses was performed by transient transfection of 293T cells as previously described [34]. For infections, cells were incubated overnight with recombinant lentiviruses. An equal amount of fresh culture medium was added 24 h later and after 48 h, cells were washed and seeded in fresh culture medium. GFP positive cells were sorted with FACSAria™ sorter (Becton Dickinson, Franklin Lakes, NJ, USA).

### 2.4. Electrophoretic Mobility Shift Assays (EMSA)

Whole cell extracts were prepared and analyzed for DNA binding activity using the HIV-LTR tandem κB oligonucleotide as κB probe [35]. Use of whole cell extracts to analyze NF-κB DNA-binding status by EMSA was previously validated by comparing nuclear and total NF-κB DNA-binding profiles and the observation of highly similar NF-κB DNA-binding activity, indicating that NF-κB binding emanates from nuclear proteins [25].

### 2.5. Measurement of Intracellular ATP Content

Intracellular ATP content was measured using a luminescence ATP detection assay kit (CellTiter-Glo^®^ Luminescent Cell Viability Assay, Promega, Madison, WI, USA) according to the manufacturer’s instructions. Briefly, 3 × 10^4^ cells were seeded in 100 µL of RPMI-1640 Glutamax medium supplemented with 10% FBS (96 well plates). Cells in triplicate were either left untreated or treated for 30 min in presence or not of 20 mM 2-deoxy-D-glucose (2-DG) to inhibit glycolysis. At the end of the incubation time, the ATP content was measured by adding 100 µL of CellTiter-Glo reaction mix to each well (final volume of 200 µL) for 10 min. The luminescence signal was recorded with a microplate reader (Centro LB 960 microplate luminometer, Berthold Technologies, Bad Wildbach, Germany). Raw data (Relative Luminescence Units) were analyzed using Microsoft Excel software (Redmond, WA, USA). The difference between total ATP content and ATP produced under 2-DG treatment results in glycolytic ATP.

### 2.6. Annexin V Binding Assay

DLBCL cells were harvested and washed twice with cold PBS. Cells were resuspended in 1X binding buffer containing either Annexin V/PE or Annexin V/APC (BD Biosciences Pharmingen, San Diego, CA, USA) and 4′,6-diaminidino-2-phenylindole (DAPI, Molecular Probes, Life Technologies, Carlsbad, CA, USA) for 15 min at room temperature. The samples were subjected to cytometric analysis with a MACSQuant Analyzer 10 Flow Cytometer (Miltenyi Biotec, Bergisch Gladbach, Germany) and the data were analyzed using the Flowjo v10.2 software (Ashland, OR, USA).

### 2.7. Measurement of Mitochondrial Transmembrane Potential (ΔΨm)

For determination of mitochondrial transmembrane potential (ΔΨm), cells were stained with 100 nM of Tetramethyl rhodamine methyl ester (TMRM) (Molecular Probes, Life Technologies, Carlsbad, CA, USA) in RPMI-1640 Glutamax medium for 30 min at room temperature. Cells were evaluated by cytometric analysis as above.

### 2.8. Cell Viability Assay

Cell viability was assessed using trypan blue dye exclusion.

### 2.9. Immunoblotting

Immunoblotting were performed as previously described [36].

### 2.10. Quantitative Real Time PCR

Total RNA extraction and reverse transcription were performed as previously described [35]. Real-time PCR analysis was carried out with LightCycler FastStart DNA Master plus SYBR Green I on a Light Cycler 1.5 (Roche Applied Science, Basel, Switzerland). All values were normalized to the level of HPRT mRNA. See Table 1 for primer sequences.

### 2.11. Statistics

Statistical significance was assessed using unpaired *t*-test (Prism 8.0, GraphPad Software, San Diego, CA, USA). A value of *p* = 0.05 was considered as statistically significant with the following degrees: * *p* < 0.05; ** *p* < 0.01; *** *p* < 0.001; **** *p* < 0.0001.

## 3. Results

### 3.1. The RelB Positive-MD901 DLBCL Cell Line Is an OxPhos-DLBCL

**Figure 1:**
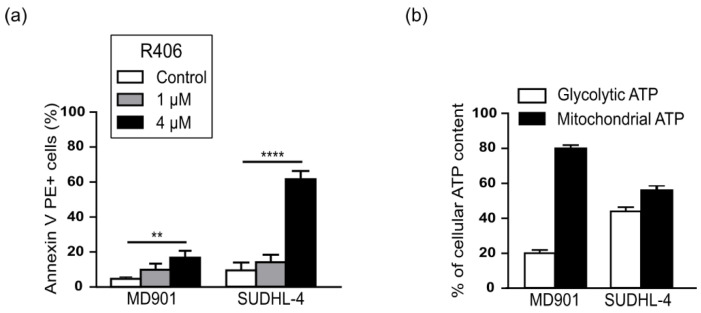
The RelB positive-MD901 DLBCL cell line is an OxPhos-DLBCL. (**a**) MD901 and SUDHL-4 DLBCL cell lines were incubated with the indicated doses of the SYK inhibitor R406 for 96 h and apoptosis was monitored by Annexin V-PE and DAPI staining followed by FACS analysis. Data are presented as mean values ± SD (*n* = 3). *p*-values by unpaired *t*-test, ** *p* < 0.01; **** *p* < 0.0001; (**b**) Intracellular levels of glycolytic and mitochondrial ATP of MD901 and SUDHL-4 cell lines was measured as described in Materials and methods.

To directly assess the contribution of RelB on DLBCL cell survival upon mitochondrial stress, we used the ABC DLBCL cell line MD901 that we have previously described as exhibiting a strong constitutive RelB DNA-binding activity [25]. Since the metabolic status of the MD901 DLBCL cell line was so far unknown, we evaluated whether this DLBCL cell line was an OxPhos or a BCR-DLBCL cell line. We first tested whether the MD901 DLBCL cell line was sensitive or not to R406, an ATP competitive inhibitor of the spleen tyrosine kinase (SYK) that only induces apoptosis in DLBCL cell lines having an active BCR signaling, whereas OxPhos-DLBCLs that do not display functional BCR signaling are insensitive to this inhibitor [16]. As presented in Figure 1a, MD901 DLBCL cell line was insensitive to R406-induced apoptosis. In contrast, as a control, the BCR-DLBCL cell line SUDHL-4 [16] was highly sensitive to R406 at the same doses. BCR-dependent DLBCL cell lines were reported to rely more on a glycolytic metabolism [15,37]. In accordance, we further demonstrated that MD901 DLBCL cells produce only a low level of glycolytic ATP, on the contrary to what was seen with the reported glycolytic BCR SUDHL-4 DLBCL cell line (Figure 1b). Altogether, our data allowed to classify the MD901 DLBCL cell line as a new OxPhos-DLBCL cell line.

### 3.2. RelB Controls Energy Homeostasis in the OxPhos MD901 DLBCL Cell Line

**Figure 2:**
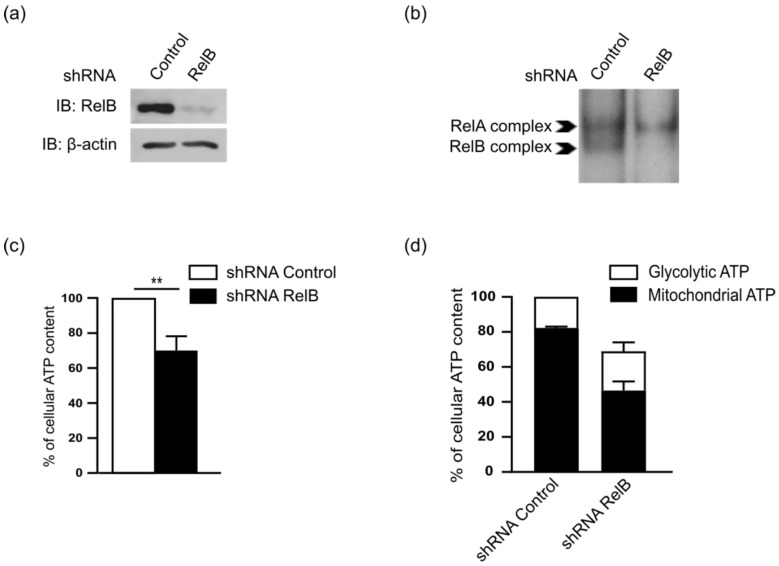
RelB controls energy homeostasis in the OxPhos MD901 DLBCL cell line (**a**,**b**) RelB protein levels (**a**) and RelB DNA binding activity (**b**) are efficiently knocked down by stable RNA interference in MD901 DLBCL cell line. Whole cell extracts from MD901 cell line transduced with lentiviruses encoding either a shRNA targeting RelB (shRNA RelB) or a scrambled control (shRNA control) were analyzed by immunoblotting (**a**) and EMSA (**b**) for the indicated proteins. (**c**) RelB knockdown decreases energy content in MD901 DLBCL cells. Total intracellular levels of ATP were measured in MD901 transduced cell lines as in (**a**). *p*-values by unpaired *t*-test, ** *p* < 0.01. (**d**) RelB knockdown decreases mitochondrial ATP levels, but not glycolytic ATP levels in MD901 DLBCL cells. Glycolytic and mitochondrial ATP levels were assessed in MD901 transduced cell lines as described in Materials and methods.

To assess, how RelB impacts on the mitochondrial metabolism, we first developed a stable RelB knockdown approach by stable RNA interference in the MD901 DLBCL cell line, using either a lentivirus carrying an shRNA targeting RelB (shRNA RelB) or a scrambled control (shRNA control). RelB protein levels were efficiently and significantly inhibited upon RelB knockdown (Figure 2a). Importantly, knockdown of RelB expression also resulted in a marked decrease in constitutive RelB binding activity without affecting RelA binding activity (Figure 2b). Strikingly, RelB inhibition markedly reduced total ATP levels under normal culture conditions (Figure 2c) without affecting the glycolytic ATP in MD901 DLBCL cells (Figure 2d). Therefore, our data indicate that RelB is critical for DLBCL cell energy production by directing metabolism towards mitochondrial ATP production.

### 3.3. RelB Protects DLBCL Cells from Mitochondrial Stress-Induced Apoptosis

**Figure 3:**
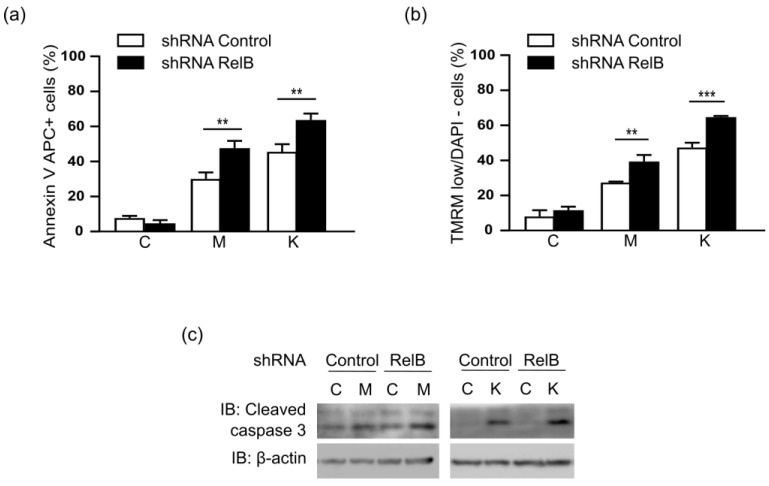
RelB protects DLBCL cells from mitochondrial stress-induced apoptosis (**a**) RelB knockdown induces MD901 DLBCL cell apoptosis upon treatment with antimetabolic drugs. MD901 cells transduced with lentiviruses encoding either a shRNA targeting RelB (shRNA RelB) or a scrambled control (shRNA control) were either left untreated or treated with either Metformin (M) (2.5 mM), or ^®^Kidrolase (K) (2 UI/mL) for 48 h, and monitored for apoptosis by Annexin V-APC and DAPI staining followed by FACS analysis. Error bars are means ± SD (*n* = 3). (**b**) RelB knockdown in DLBCL cells induces loss of mitochondrial transmembrane potential (ΔΨm) upon treatment with antimetabolic drugs. MD901 DLBCL cells transduced as in (**a**) and either left untreated or treated with either Metformin (M) (2.5 mM), or ^®^Kidrolase (K) (2 UI/mL) for 36 h were monitored for loss of mitochondrial transmembrane potential (ΔΨm) by TMRM staining followed by FACS analysis. Error bars are means ± SD (*n* = 3). *p*-values by unpaired *t*-test, ** *p* < 0.01; *** *p* < 0.001. (**c**) RelB knockdown in DLBCL cells induces caspase 3 cleavage upon treatment with antimetabolic drugs. MD901 DLBCL cells transduced as in (**a**) and either left untreated or treated with either Metformin (M) (2.5 mM), or ^®^Kidrolase (K) (2 UI/mL) for 8 h were monitored by immunoblotting for the indicated proteins.

Since RelB controls overall mitochondrial ATP content, we next assessed the contribution of RelB in the DLBCL cell survival upon mitochondrial metabolic stress. Therefore, we next treated MD901 cells with either Metformin, or L-asparaginase (^®^Kidrolase), two antimetabolic FDA approved drugs targeting the mitochondrial metabolism. Metformin is a widely prescribed anti-hyperglycemic biguanide drug to type II diabetics, inhibiting specifically complex I of the mitochondrial electron transport chain [38]. L-asparaginase (^®^Kidrolase) catalyzes the hydrolysis of asparagine and glutamine into aspartate and glutamate, respectively, accompanied by mitochondria dysfunction [39,40]. Metformin and Kidrolase both induced significant apoptosis in MD901 DLBCL cell line as evaluated by Annexin V/DAPI staining (Figure 3a) Most importantly, RelB-expression knockdown markedly increased either Metformin or ^®^Kidrolase induced apoptosis. (Figure 3a). This effect was associated with an increase in loss of mitochondrial potential (ΔΨm) (Figure 3b), and cleavage of caspase 3 (Figure 3c). Collectively, our findings demonstrate that RelB inhibition enhances the pro-apoptotic efficacy of anti-metabolic drugs targeting mitochondrial metabolism.

### 3.4. RelB protects DLBCL Cells from Induction of Apoptosis upon Glutamine Starvation

**Figure 4:**
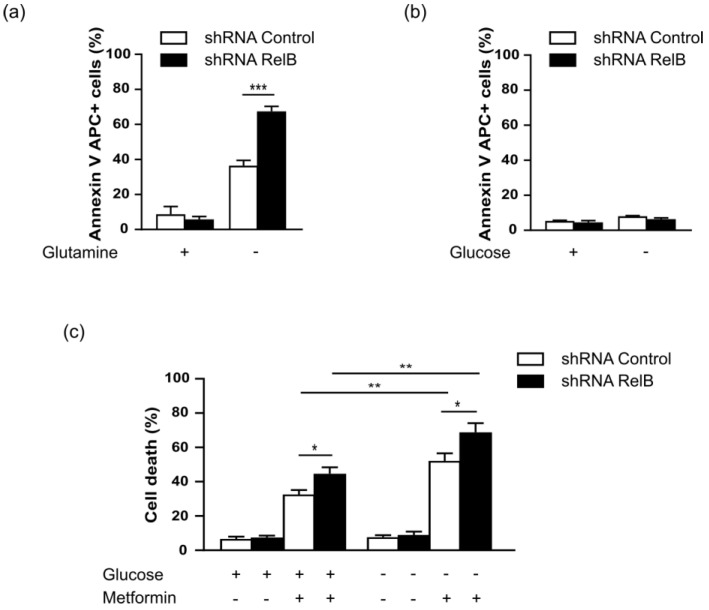
RelB inhibition enhances sensitivity to cell apoptosis upon glutamine deprivation. RelB protects DLBCL cells from induction of apoptosis upon glutamine deprivation (**a**,**b**) MD901 cells transduced with lentiviruses encoding either a shRNA targeting RelB (shRNA RelB) or a scrambled control (shRNA control) were cultured for 48 h in either glutamine-free (**a**) or glucose-free cell culture medium (**b**), or in normal cell culture medium containing glutamine and glucose (**a**,**b**), and monitored for apoptosis by Annexin V-APC and DAPI staining followed by FACS analysis. Data are presented as mean values ± SD (*n* = 3). *p*-values by the unpaired *t*-test, ** *p* < 0.01; *** *p* < 0.001. (**c**) MD901 DLBCL cells transduced as in (**a**), were either treated by Metformin (2.5 mM) or left untreated in either normal cell culture medium containing glucose or in glucose-free cell culture medium and monitored for cell viability by trypan blue exclusion. +: presence; −: absence. Data are presented as mean values ± SD (*n* = 3). *p*-values by the unpaired *t*-test, * *p* < 0.05; ** *p* < 0.01.

Cancer cells show abnormal glucose and glutamine metabolism for survival advantages [41,42,43]. Therefore, we investigated the impact of the lack of these nutrients on DLBCL cell survival and the contribution of RelB in this context. We observed that glutamine depletion induced a marked apoptosis, thus showing that glutamine metabolism is essential for MD901 DLBCL cell survival (Figure 4a). Strikingly, glutamine starvation-induced apoptosis was markedly increased upon knockdown of RelB expression (Figure 4a). In contrast, no significant apoptosis was induced upon glucose deprivation in MD901 DLBCL cells (Figure 4b), and RelB expression inhibition has no further impact on the DLBCL cell survival upon glucose starvation (Figure 4b). Altogether, our data indicate that glutamine, but not glucose, is critical for OxPhos-DLBCL cell survival, and that RelB exerts a crucial pro-survival function in DLBCL cells upon glutamine deprivation.

To go one step further, we wanted to see how glucose may impact on DLBCL cell survival upon mitochondrial metabolic stress. Therefore, we evaluated the impact of Metformin on DLBCL cell survival upon glucose deprivation. As seen on Figure 4c, MD901 cells were much more sensitive to cell death induced by Metformin upon glucose deprivation, indicating that glucose is an important nutrient for DLBCL resistance to Metformin-induced cell death. Strikingly, knockdown of RelB expression led to an even more pronounced cell death induced by Metformin treatment upon glucose starvation (Figure 4c), showing that RelB inhibition renders DLBCL MD901 cells highly susceptible to metabolic challenge.

### 3.5. RelB Inhibition Decreases Oxidative Stress Response- and Anti-Apoptotic-Gene Expression upon Anti-Metabolic Drug Treatment in MD901 DLBCL Cells

**Figure 5:**
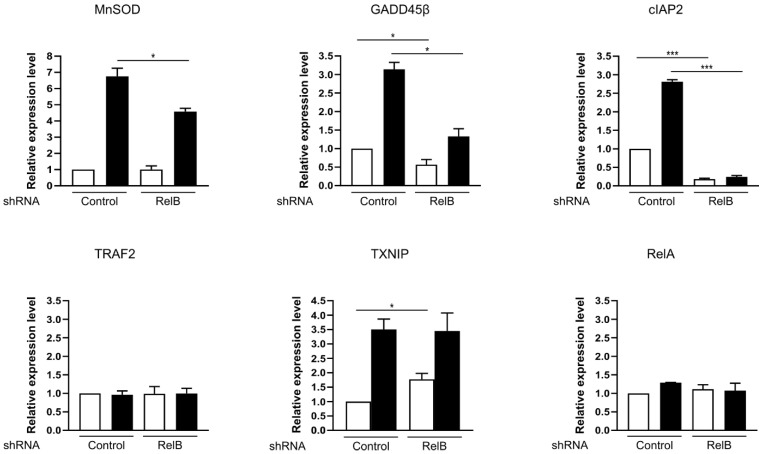
RelB inhibition decreases oxidative stress response- and anti-apoptotic-gene expression upon anti-metabolic drug treatment in MD901 DLBCL cells. Quantitative RT-qPCR was performed with specific primer pairs for the indicated genes using total RNAs prepared from MD901 cells transduced with lentiviruses encoding either a shRNA targeting RelB (shRNA RelB) or a scrambled control (shRNA control), and either left untreated or treated by Metformin (M) (2.5 mM) combined with ^®^Kidrolase (K) (2 UI/mL). Results are means ± SD of two independent experiments normalized to the level of HPRT mRNA. *p*-values by unpaired *t*-test: * *p* < 0.05; *** *p* < 0.001.

Since RelB-dependent metabolic pathway in MD901 DLBCL cells involves stimulation of OxPhos (Figure 2c,d), and RelB impacts on DLBCL survival upon the treatment with antimetabolic drugs (Figure 3a–c), we next examined whether RelB activation in DLBCL cells upregulates the expression of endogenous NF-κB responsive genes involved in oxidative/metabolic stress response and survival upon combination of these two antimetabolic drugs. Knockdown of RelB expression markedly and significantly decreased MnSOD (Manganese superoxide dismutase), GADD45β (Growth arrest and DNA damage inducible beta) and cIAP2 (Cellular inhibitor of apoptosis protein 2) mRNA levels upon Metformin and Kidrolase treatment (Figure 5). In contrast, no differences in TRAF2 (TNF receptor associated factor 2), TXNIP (Thioredoxin interacting protein) and RelA mRNA levels were observed upon RelB knockdown. Taken together, these results indicate that RelB activation in OxPhos-DLBCL cells is associated with increased expression of a subset of NF-κB target genes involved in oxidative stress response (MnSOD), inhibition of metabolic stress-induced cell death (GADD45β) and extrinsic mitochondrial apoptotic pathway of survival (cIAP2) upon antimetabolic treatment.

## 4. Discussion

Although recent evidence support the ability of the classical NF-κB to regulate energy homeostasis and cancer cell metabolic reprogramming [32,33,44,45], a role for the alternative NF-κB pathway in cancer cell metabolism remains almost undefined. In the study presented here, we conducted the first analysis of the involvement of the alternative RelB NF-κB subunit in the control of energy homeostasis and response to antimetabolic drugs in DLBCL cells. We revealed that RelB constitutive activation in OxPhos-DLBCL cells programs DLBCL cells towards oxidative energy metabolism, indicating that RelB impacts on energy metabolism in these cancer cells. Moreover, we uncovered an important role for RelB in protecting DLBCL cells from apoptosis induced by treatment with antimetabolic drugs targeting mitochondrial metabolism. Even though the impact of RelB on DLBCL metabolism should be evaluated on a larger panel of DLBCL cell lines, nonetheless these results have important implications for the role of RelB in DLBCL pathogenesis and treatment.

Our study has revealed that RelB inhibition causes a decrease in cellular ATP levels by reducing mitochondrial ATP production without affecting glycolytic ATP levels in a RelB-positive OxPhos-DLBCL cell line. Accordingly, RelB knockdown did not induce a significant spontaneous DLBCL cell apoptosis upon glucose starvation. Thus, our results suggest that DLBCL cell metabolism does not reprogram towards aerobic glycolysis upon RelB inhibition in RelB-positive OxPhos-DLBCL cells, which is worth further investigation. Nonetheless, RelB upregulates mitochondrial metabolism in DLBCL cells.

Rewiring of cancer cell metabolism is now recognized as a hallmark of cancer [27] and represents a key cause of resistance to current anticancer therapeutic armorentum. Therefore, targeting tumor energy metabolism has emerged as a very promising strategy to overcome resistance to treatment. Our study shows that RelB protects DLBCL cells from mitochondrial metabolic stress. First, RelB knockdown induces a marked increase in DLBCL cell apoptosis upon treatment with Metformin, a drug that inhibits the complex I of the mitochondrial electron transport chain [38]. Second, upon RelB expression inhibition, we observed an increased sensitivity of DLBCL cell apoptosis induced by glutamine deprivation, a central amino acid for the replenishment of the TCA cycle intermediates. Third, RelB exerts a critical protective role upon treatment with the drug L-asparaginase (Kidrolase) that also exhibits a strong extracellular glutaminase activity [39,40]. Fourth, RelB activity is required for optimal expression of NF-κB target genes involved in oxidative stress response (MnSOD), inhibition of metabolic stress-induced cell death (GADD45β) and extrinsic mitochondrial apoptotic pathway of survival (cIAP2) upon antimetabolic treatment, although it remains unknown if the pro-survival activity of RelB is exerted through the control of the expression of these genes. Beyond DLBCL, close relation between oxidative stress and RelB activity has also been reported in prostate cancer cells [46,47]. Thus, it is likely that the pro-survival effects of RelB upon metabolic stress may be generalized to other neoplasms, especially those addicted to alternative NF-κB.

In conclusion, we established that RelB plays an important role in the maintenance of DLBCL cell energy metabolism and is a crucial positive regulator of DLBCL cell survival upon mitochondrial stress. These findings have therapeutic implications as they indicate that alternative NF-κB inhibitors may enhance the anticancer efficacy of antimetabolic drugs, thus providing a strong rationale for the development of new molecules targeting RelB for therapeutic intervention in cancer.

## Figures and Tables

**Table 1:** List of primer sequences used for RT-PCR analysis. F: Forward primer. R: reverse primer.

Genes	Primer Sequences
MnSOD	F: 5′-TGTGCTTTCTCGTCTTCAGC-3′ R: 5′-GAGCCCAGATACCCCAAAG-3′
GADD45β	F: 5′-GCCAGCTACTGCGAAGA-3′ R: 5′-TGTTTGTGGCAGCAACTCAAC-3′
cIAP2	F: 5′-ACTAATACCGGGAACA-3′ R: 5′-ACTCCTGGGCTCAAGTAATTC-3′
TRAF2	F: 5′-GCATACCCGCCATCTTCTC-3′ R: 5′-CGTTCAGGTAGATACGCAGACA-3′
TXNIP	F: 5′-CTTCTGGAAGACCAGCCAAC-3′ R: 5′-GAAGCTCAAAGCCGAACTTG-3′
RelA	F: 5′-TTGAGCCCACAAAGCCTTATCAAGT-3′ R: 5′-GGACAATGCCAGTGCCATACAG-3′
HPRT	F: 5′-GGCGTCGTGATTAGTGATG-3′ R: 5′-GCACACAGAGGGCTACAATGT-3′

## Data Availability

Data are all contained within the article.
